# Comparing the Antifungal Effect of Sodium Hypochlorite Gel versus Different Types of Root Canal Medicaments at Different Time Intervals Using the Agar Diffusion Test: An *In Vitro* Study

**DOI:** 10.1155/2021/6550054

**Published:** 2021-12-13

**Authors:** Mohamed El Sayed, Nikta Ghanerad, Zeinab Shabanpour, Mahin Shabanpoor, Fatemeh Rahimi

**Affiliations:** ^1^Ajman University, College of Dentistry, Department of Clinical Sciences, P.O. Box 346, Ajman, UAE; ^2^Mansoura University, Faculty of Dentistry, Department of Endodontics, El-Gomhoureya Street, El-Mansoura 35516, El-Dakahleya, Egypt

## Abstract

**Aims:**

The purpose of this study was to compare the antifungal activity of sodium hypochlorite gel to those of four intracanal medicaments at various time intervals.

**Materials and Methods:**

The agar well diffusion technique was utilized to test the antifungal activity of the following medicaments against *Candida albinans* (*C. albicans*): sodium hypochlorite gel, chlorhexidine gel, calcium hydroxide paste, Ledermix, and Diapex Plus. The inhibition zone related to each medicine was measured in millimeter after 24, 48, and 72 hours of incubation at 37°C. The data were analyzed using one-way ANOVA and Games–Howell tests, at a significance level of *P* < 0.05.

**Results:**

Except for Ledermix and Diapex Plus, which had no antifungal action, all of the medicaments showed varied inhibitory zones for *C. albicans*. At all periods, the NaOCl gel had the most significant inhibition zones, followed by the CHX gel and calcium hydroxide. At all time intervals, the NaOCl gel demonstrated comparable antifungal efficacy. When compared to a 24-hour time interval, the CHX gel showed an increased antifungal activity at the 48-hour and 72-hour intervals. Calcium hydroxide, on the other hand, showed a decrease in its antifungal activity at the 72-hour interval.

**Conclusion:**

The antifungal activity of sodium hypochlorite gel was the highest among the investigated medicaments. Chlorhexidine gel's antifungal activity improved over time, whereas calcium hydroxide's antifungal activity declined. Diapex Plus and Ledermix did not have any antifungal properties.

## 1. Introduction

Microorganisms are thought to be the primary cause of pulpal and periapical diseases [1]. Microbial contamination or infected root canals may delay periapical lesions healing and reduce the endodontic treatment success rates [2]. Resistant bacteria might survive as a result of poor biomechanical instrumentation, inappropriate root canal filling, or reinfection by the microleakage process [3]. These microorganisms are frequently responsible for the endodontic failure and the development of chronic periapical lesions [4].

For a long time, Grossman realized that the existence of fungi within the infected root canals might complicate the endodontic treatment process. These fungi must be eliminated using intracanal medicaments with a proper antifungal activity [5]. Several studies supported this concept and found that fungi play an imperative role in the development of pulpal and periapical infections, as well as root canal treatment failure [6–8].

The primary objective of root canal therapy is to eliminate the microorganisms and their byproducts from the infected radicular system while also preventing the regrowth of dormant ones [9]. Unfortunately, the chemomechanical preparation of the root canals is unable to eliminate all radicular microorganisms because of the intricacy of the root canal anatomy [10] and the deep penetration of microbes within the dentinal tubules [11]. As a result, the intracanal medicaments become more important, particularly in cases when the infection is resistant to the traditional endodontic treatment and when the treatment's success is challenged.

Various intracanal medicaments, such as nonsetting calcium hydroxide (Ca(OH)_2_) and chlorhexidine gluconate (CHX) gel, were clinically and laboratory tested against many types of microorganisms, and they showed variable levels of antimicrobial effect [12–15]. Ledermix is a popular intracanal medication with a polyethylene glycol base that contains 1% triamcinolone and 3.2% demeclocycline. It has an anti-inflammatory effect because of the presence of corticosteroid (triamcinolone), and hence, it can be used to minimize pain or discomfort accompanying symptomatic apical periodontitis and to avoid symptomatic apical periodontitis flare-ups [16]. The antibiotic component of Ledermix (demeclocycline) counteracts the corticosteroid's suppression of the local immune response and minimizes the risk of infection [16]. Diapex Plus (DiaDent, Seoul, Korea) is yet another intracanal medication that has recently launched as a premixed paste consisting of 40.4% iodoform, 30.2% calcium hydroxide, and 22.4% silicone oil. The addition of iodoform to the calcium hydroxide paste is supposed to enhance its antibacterial properties [17]. Diapex Plus has a significant antibacterial action according to the manufacturer and can be used to treat pulpal and periapical infections.

The most frequent root canal irrigating solution is sodium hypochlorite that has antibacterial and tissue-dissolving characteristics [18]. It has been proven that 0.5% NaOCl kills *C. albicans* after a 10-second contact period [18]. However, when apically extruded, this solution causes significant tissue toxicity [18]. As a result, using NaOCl in a gel form reduces the risk of apical extrusion and the unfavorable consequences of the solution form [19, 20]. Sodium hypochlorite (NaOCl) gel is currently available under the name of bleaching pen (Clorox Bleach Pen, USA) to clean precisely the dirty white clothes. The composition of that Bleach Pen is 0.5–2% sodium hypochlorite, 3–7% boehmite, 0.5–1.5% sodium silicate, and 0.5–1.5% sodium petroleum sulfonate. However, the vendor has kept the exact concentration of sodium hypochlorite a trade secret [21]. Elsayed *et al.* [22] compared the antibacterial activities of the Clorox Bleach Pen to those of other commonly used intracanal medications. The authors found that the Clorox Bleach Pen had the most potent antibacterial effect, and they recommended its use as an intracanal medicament.

Endodontic materials can be examined *in vivo* or *in vitro* for their antibacterial properties. The most common method for testing the antibacterial activity of a specific dental material is still the agar-well diffusion method [23]. This technique is straightforward to conduct and is of low cost, and it assures that the chemical properties of the materials being examined are conserved [24].

Many studies investigated the bactericidal and fungicidal properties of the presently available intracanal medicaments with inconsistent and incomplete results. Furthermore, the antifungal activity of sodium hypochlorite gel has never been studied to the best of our knowledge. Therefore, the goal of this study was to examine and compare the antifungal efficacy of sodium hypochlorite gel to that of calcium hydroxide paste, chlorohexidine gel, Ledermix, and Diapex Plus against *C. albicans* using the agar well diffusion method at different time intervals. The null hypothesis for this study is that the antifungal effects of all examined medicaments against *C. albicans* are similar.

## 2. Materials and Methods

### 2.1. Experimental Intracanal Medicaments

The following experimental medicaments were evaluated (Table 1): sodium hypochlorite gel (Clorox, Bleach pen Gel for white, USA), 2% chlorhexidine gel (Conspsis Scrub, Ultradent Products, USA), nonsetting calcium hydroxide paste (Metapast, Meta Biomed com., LTD., Korea), Ledermix (Riemser, Germany), and Diapex Plus (DiaDent, Korea).

### 2.2. Experimental Microorganism

*C. albicans* ATCC 10231 (Microbiologics, USA) was utilized to investigate the inhibitory characteristics of the experimental medicaments.

### 2.3. Inoculum Suspension Preparation

*C. albicans* was cultured aerobically in Sabouraud Glucose broth (Difco Laboratories, Detroit MI, USA) for 48 hours at 37°C. The cultured broth was diluted in 0.85% sterile saline solution to obtain an experimental colony suspension (Inoculum suspension) with 0.5 turbidities on the McFarland scale (1.5 × 108 fungi/mL).

### 2.4. Agar Well Diffusion Test (AWDT)

Agar well diffusion tests were performed on Petri dishes (agar plates) with a 90 mm diameter and 4 mm depth of Sabouraud Glucose agar (Merck KGaA, Darmstadt, Germany). Two plates were used to assess the antifungal effect of the experimental root canal medicaments against *C. albicans*. Using disposable inoculating loops (Merck KGaA, Darmstadt, Germany), the top surfaces of the agar plates were inoculated with 100 *µ*l of inoculum suspension and then air-dried for 15 minutes at 37°C. Two wells of 5 mm diameter and 4 mm depth were cut in the first agar plate with a sterile glass Pasteur pipette, while three wells of identical diameters and depths were cut in the second agar plate. The distance between the wells was standardized to be 30 mm. A sterilized pipette was also utilized to place 60 *μ*L of each medication into its agar plate well. The wells in the first agar plate were filled individually with sodium hypochlorite gel and calcium hydroxide paste, while the wells in the second agar plate were filled individually with Diapex Plus, Ledermix, and chlorohexidine gel. The experimental plates were preserved at room temperature for 2 hours to allow the diffusion of the tested medicaments into the agar media before being incubated at 37°C for 72 hours under aerobic conditions. For optimal reliability of the results, the whole experiment was repeated ten times.

### 2.5. Preparing Negative and Positive Growth Controls (Figure 1)

#### 2.5.1. Negative Growth Control (Three Agar Plates)

Three noninoculated agar plates were prepared the same as the experimental agar plates. The selected medicaments were placed in the wells of two plates, while the agar wells of the third plate were left empty.

#### 2.5.2. Positive Growth Control (Two Agar Plates)

Two agar plates without medicaments were streaked with *C. albicans* to guarantee that their lifecycle was not disrupted during the experiment.

### 2.6. Measuring the Diameter of Inhibitory Zones (Figure 2)

The lack of fungal colonization close to each agar well (agar clearing) demonstrated the growth inhibition zones around each medicament. The most consistent diameter of each inhibitory zone, including the diameter of the agar wells (6 mm), was identified and measured in millimeters using an endodontic metal ruler (Hu-Friedy Mfg., USA). The diameter of the growth inhibition zones was measured at 24, 48, and 72-hour intervals. All measured values beyond the agar-well diameter indicated a significant inhibition for the growth of *C. albicans* and a greater antifungal activity of the tested medicaments. Finally, ten measurements were taken for each medicament at each time interval after the experiment was completed.

### 2.7. Statistical Analysis

The data were statistically evaluated using SPSS software version 20 (IBM Corporation 1 New Orchard Road Armonk, New York, USA). The mean diameters of the inhibition zones around each medicament were compared at each time interval using the one-way ANOVA and Games–Howell post hoc testing. Furthermore, the mean diameters of inhibition zones at 24, 48, and 72 hours for each medicament were compared. The statistical significance was determined when the *P* value is less than 0.05.

## 3. Results

**Figure 1:**
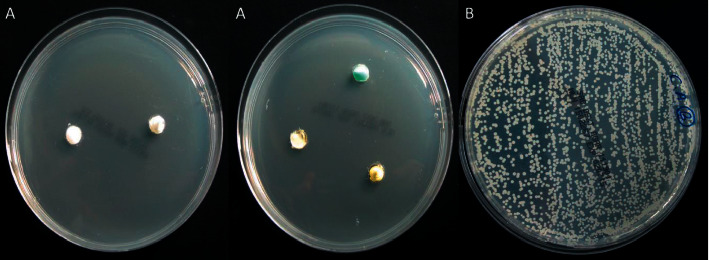
Agar plates for growth control: (a) negative growth control and (b) positive growth control.

**Figure 3:**
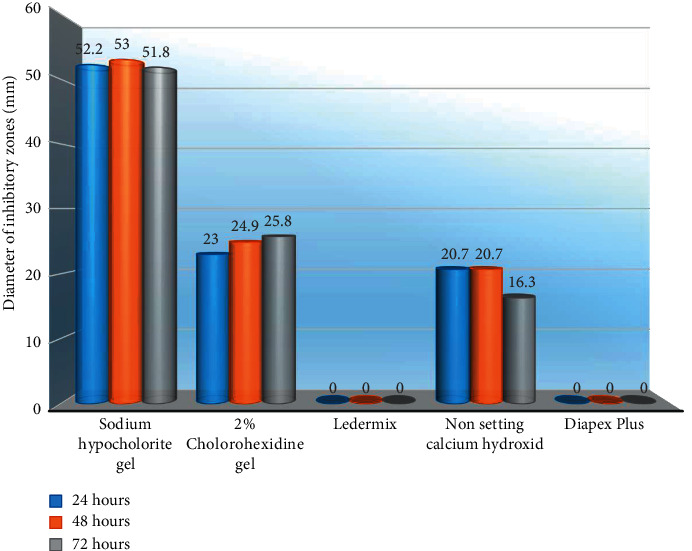
Assessment of the antifungal effect of sodium hypochlorite gel, 2% chlorhexidine gel, Ledermix, Calcium hydroxide, and Diapex Plus against *C. albicans* at different time intervals.

**Figure 4:**
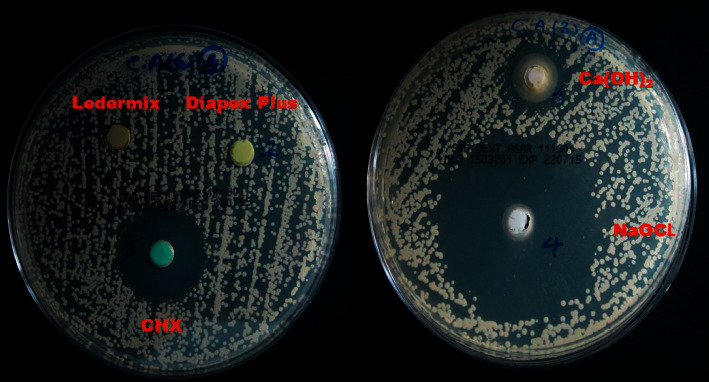
Zones of growth inhibition of C. *albicans* on Saboroud agar plates that were created by the tested intracanal medicaments.

The mean values of the inhibition zones for each medicament against *C. albicans* at all time intervals are given in Table 2 and represented in Figure 3. The negative control plates showed no fungal growth, while the positive control plates revealed obvious and even fungal growth (Figure 1(b)). At all time intervals, sodium hypochlorite gel showed the strongest antifungal activity. Except for Ledermix and Diapex Plus, which had no inhibitory effects on *C. albicans,* the other medicaments had varying antifungal effectiveness. However, it was substantially lower than that of the sodium hypochlorite gel (Figure 4). The antifungal activity of sodium hypochlorite at all time intervals was nearly similar (*P* > 0.05). The antifungal activity of CHX gel increased after 24 hours, while the antifungal activity of nonsetting calcium hydroxide paste decreased after 48 hours.

## 4. Discussion

The use of antimicrobial medicaments between appointments is required to disinfect the root canal system and improve the outcome of endodontic treatment [12]. As the fungi have been linked to the cases of persistent and secondary periapical infections [13], the intracanal medicaments should have an adequate antifungal activity [25]. Therefore, the present study aimed to investigate the antifungal efficacy of the following medicaments: calcium hydroxide (Metapaste), 2% chlorohexidine gel (Conspsis Scrub), antibiotic-corticosteroid mixture (Ledermix), calcium hydroxide-Iodoform mixture (Diapex Plus), and sodium hypochlorite gel (Clorox, Bleach Pen Gel). The majority of intracanal medications lose their antimicrobial activity after twenty-four hours, and they are completely ineffective after 72 hours [26]. Consequently, the present study evaluated the antifungal effectivity of the tested medicaments after twenty-four, forty-eight, and seventy-two hours.

Calcium hydroxide paste was selected in this study as it has a long history of antimicrobial properties and the ability to stimulate mineralization [27]. However, there are controversies about its antifungal effect against *C. albicans* [28–30]. Furthermore, even after continuous contact with the root canal walls, some investigators have questioned its usefulness in lowering the bacteria levels [31]. Antimicrobial agents with specific chemical characteristics have been suggested to be used as vehicles with calcium hydroxide to enhance its antimicrobial effect [32]. The potent bactericidal properties of iodoform paste have been established in previous studies [33]. Some authors found that iodine has both bactericidal and fungicidal effects [34]. Moreover, iodine has reasonable tissue biocompatibility and is often used as a resorbable dressing in the pulpectomies of infected deciduous teeth [34, 35]. There is insufficient evidence concerning the antifungal activity of Diapex Plus. As a result, Diapex Plus, a calcium hydroxide/iodoform combination in an oily vehicle, was chosen as one of the medicaments to be examined in the current study. Some authors demonstrated that the addition of oily vehicles to calcium hydroxide improves its antimicrobial effects [36].

Another root canal medication used in this study was chlorhexidine gel. It has good antibacterial and antifungal properties [37]. In addition, it does not affect the root canal's apical seal [38]. Some researchers proposed using 2% chlorhexidine gel as a root canal medicament rather than calcium hydroxide [39]. Earlier findings have proved the efficacy of Ledermix as an intracanal medicament [40, 41]. Demeclocycline calcium and triamcinolone acetonide are the active ingredients of the Ledermix past. There were controversies regarding its broad-spectrum antimicrobial effect [42, 43]. In addition, there is secrecy in the studies about the antifungal properties of Ledermix. Hence, it was selected in the present study to test its antifungal efficacy.

Looking for an effective intracanal medicament against *C. albicans* is currently required. NaOCl is considered the most popular irrigating solution that had a strong antimicrobial action against most endodontic microorganisms [44]. Besides, it has an excellent dissolving effect on vital and necrotic tissues [45]. However, this solution is highly toxic at high concentrations if it is extruded apically [46]. The use of of NaOCl gel as an irrigating solution was suggested by some authors to overcome the problem of apical extrusion of its solution form [19, 20]. To the best of our knowledge, no dental company produced NaOCl gel as a root canal medicament. However, some detergent companies such as Clorox (Clorox, Oakland, CA 94612, USA) introduced the gel form of NaOCl as a bleaching agent for white clothes. As a result, the antifungal effect of NaOCl gel (Clorox Bleach Pen) as a trial root canal medicament was tested in this study.

The agar diffusion technique was chosen in the current research to evaluate the antifungal effect of the selected medicaments since it is a straightforward technique and can be used as a preliminary test before performing more sophisticated tests [23, 24, 47]. By evaluating the size of the developed inhibitory zones, this methodology allows the direct comparisons of endodontic materials' antifungal activity, revealing which material can remove the potential pathogens within the root canal system [48]. However, the size of the inhibitory areas, on the other hand, does not reflect the entire performance of the investigated substance [49]. Many factors, including the chemical and physical qualities of the examined material, as well as the culture media, can influence the results [50]. One of the agar diffusion test's drawbacks is its inability to distinguish between microbial growth inhibition and total microbial death [51]. In the current investigation, all attempts were undertaken to standardize many variables, such as agar medium type and thickness, inoculum density, and incubation temperature [48]. Sabouraud's glucose agar was used as the culture medium since this media is readily available and selectively used for *C. albicans* [26].

The null hypothesis of the current investigation was rejected since the results revealed significant differences in the inhibitory effect of the tested medicaments on *C. albicans* growth.

The current findings revealed that NaOCl gel had the highest antifungal activity (51.8 mm inhibition zone after 72 hours) among the tested medicaments at all time intervals. The chlorhexidine gel showed approximately 50% less antifungal activity (25.8 mm inhibition zone after 72 hours) than the NaOCl gel. Nejad Shamsi et al. [52] concluded that the solution and gel forms of NaOCl exhibited similar antibacterial effects against *E. faecalis*, and the NaOCl gel can be recommended as an efficient intracanal irrigation agent. Some authors [53, 54] found that NaOCl solution at low concentrations can destroy *C. albicans* within a few minutes, and they suggested this solution as a denture cleanser. The present results support those of White et al. [55], who found that using chlorhexidine irrigation solution might prevent dentine reinfection for up to 72 hours, and according to other authors [56], for several weeks. Ruff et al. [57] showed that the effectiveness of 2% CHX and 6% NaOCl against *C. albicans* was equal, and those results are not matching with the current results. The cause of this disagreement may be due to the use of NaOCl and CHX in their solution forms and the differences in the assessment method of their antifungal effect.

The antifungal property of sodium hypochlorite may be explained by the release of hypochlorous acid when it is mixed with water. This acid has an active chlorine, a powerful oxidizing agent that permanently oxidizes the –SH groups of essential metabolic enzymes, thereby affecting the metabolic processes of microorganisms [58]. The elimination of the smear layer before using NaOCl could enhance its antifungal activity of intracanal medicaments against *C. albicans* [59]. Despite its powerful antibacterial and antifungal properties, one of the major limitations of NaOCl in the root canal system is its high surface tension, which prevents it from penetrating dentinal tubules and other inaccessible areas of the root canal system [60]. Therefore, further studies should be done on the surface tension of NaOCl gel and its ability to penetrate the dentinal tubules. Also, the substantivity of NaOCl gel should be evaluated.

In the current study, the antifungal effects of chlorhexidine gel and calcium hydroxide paste are comparable at 24- and 48-hour intervals. However, after 72 hours, CHX gel showed a significantly higher antifungal effect than calcium hydroxide. These results are partially matching with the results of Mozayeni et al. [61] who did not find a major difference between the antifungal effects of calcium hydroxide and CHX after 24 hours. However, the same authors [61] did not find a significant difference between the antifungal effects of calcium hydroxide and CHX after 72 hours and after seven days. These results are conflicting with the current results. This disagreement may be because of the differences in the preparation of calcium hydroxide and CHX and the methods of testing. However, the present results are consistent with the results of Bellal et al. [26], who showed that chlorohexidine had better antifungal efficacy than calcium hydroxide. The current results support the finding of Vaghela et al. [15], who demonstrated that CHX had a potent fungicidal effect even at deep levels of the dentinal tubules. Several studies corroborated the current findings regarding the superiority of 2% chlorhexidine gel over calcium hydroxide in terms of their antifungal effects [62–64].

The better antifungal effect of CHX can be explained by its substantivity [65] and its high diffusion ability into the agar medium [66]. The antimicrobial activity of CHX is because of the binding of its positively charged molecules to the negatively charged areas on the microbial cell wall, causing it to be disrupted [67]. After damaging the cell wall, chlorhexidine penetrates the microbial cell and damages the cytoplasmic membrane, allowing the cell components to flow out and the microbial cell to die [68]. Based on a review of the literature on the use of chlorhexidine in dentistry, Fardal and Turnbull [69] found that chlorhexidine has a bacteriostatic effect at low concentrations and a bactericidal effect at high levels.

In this study, the effect of calcium hydroxide against *C. albicans* was high at 24 and 48 hours. It then decreased significantly at 72 hours. These observations might be explained by the role of the calcium ions in the regulation of *C. albicans* morphogenesis [29]. The release of calcium ions initially inhibited the mycelial growth of *C. albicans* [42]. The initial high pH of calcium hydroxide and its dissociation into the highly interactive and lethal hydroxyl ions kill the bacterial cells by destroying the cytoplasmic membrane and denaturing the protein and DNA [7]. Nevertheless, with the further release of calcium ions in the surrounding environment, *C. albicans* will regrow [42]. Even though *C. albicans* is highly resistant to calcium hydroxide *in vitro* [13], some researchers claimed that it can be eliminated from bovine dentin after seven days of calcium hydroxide application [70]. However, the long-term usage of calcium hydroxide may make the radicular dentin more brittle, increasing the likelihood of future cervical root fractures [71]. Some authors showed the high resistance of *C. albicans* to calcium hydroxide medication [30], and Zancan et al. [72] demonstrated that calcium hydroxide is not an effective intracanal medication against microbial biofilms. The buffering agents in the culture medium, which increase with time, may decrease the antifungal effect of calcium hydroxide [73]. However, Ferguson et al. [74] demonstrated that the calcium hydroxide paste was particularly effective against *C. albicans.* The combination of calcium hydroxide with chlorhexidine failed to reduce *C. albicans* from infected dentin even after one-week application [75]. The antibacterial activity of hydroxyl ions is also attributed to the formation of a strongly alkaline medium that causes the degradation of lipids, the major constitutes of the bacterial cell membrane, as well as structural damage to bacterial proteins and nucleic acids [76]. The present findings showed that calcium hydroxide had a lower antifungal effect in comparison with NaOCl gel and CHX gel. This may be explained by its delayed dissociation [77] and the proton pump property of the microorganisms [13]. Furthermore, the acidic pH of the culture medium utilized in this study [72] may have a buffering effect on calcium hydroxide, reducing its antifungal activity [78].

Despite combining iodoform with calcium hydroxide and forming an oily paste to enhance its antimicrobial efficacy, the current findings show that this medication has no antifungal effect against *C. albicans*. The present results are consistent with those of Gautam et al. [79], who found that Metapex, which has a similar structure to Diapex Plus, had no antifungal effect. Furthermore, several studies showed that Metapex had weak antimicrobial activity [80–82]. The oily base of Diapex Plus may prevent the release of calcium and hydroxyl ions that are necessary to destroy *C. albicans* [83]. Estrela et al. [83] showed that the addition of iodoform did not increase the antimicrobial effect of calcium hydroxide medicament. Others concluded that using nonaqueous mixing vehicles diminished the efficacy of calcium hydroxide as an intraarticular dressing [84].

Ledermix is a corticosteroid-antibiotic paste that contains 1% triamcinolone and 3.2% demeclocycline calcium in a polyethylene glycol base [85]. Abbott et al. [86] revealed that the concentration of demeclocycline within Ledermix paste is effective enough against specific bacteria. However, in the present study, it had no antifungal effect, which maybe because of the low solubility of demeclocycline calcium [87]. Furthermore, the tetracycline products have bacteriostatic effect but no antifungal impact [12, 88, 89]. MackNeill et al. [88] confirmed that tetracycline hydrochloride has no antifungal effect. According to several researchers, demeclocycline calcium has lower antibacterial activity than calcium hydroxide [90]. The current findings differ from those of Athanassiadis et al. [47] and Chua et al. [91] who found that Ledermix paste has a good antifungal impact against *C. albicans*. The difference in the methodology of those laboratory studies could be the source of this conflict.

The efficacy of any intracanal medication to disturb or kill microorganisms in a biofilm structure is more important than the resistance of a certain microorganism to that medication [92]. It must be considered that the antimicrobial adequacy of irrigating solutions and medicaments *in vitro* may be very distinctive when compared to the blended cultures present in an energetic biological structure as ordinarily happens *in vivo*. Thus, the antifungal efficacy of the tested intracanal medicaments on fungal biofilm needs further studies.

Dental companies should consider the current findings of NaOCl gel and improve its composition so that it can be utilized as an intracanal medicament. Until more laboratory and clinical investigations on NaOCl gel, CHX gel and calcium hydroxide are considered the principal intracanal medicaments that can be administered efficiently and safely. The use of Ledermix or Diapex Plus is not advocated in cases with severe or persistent endodontic infections.

## 5. Conclusions

Within the limitations of the current study, the following conclusions can be drawn:

1. Sodium hypochlorite gel had the strongest antifungal activity of all the medicaments tested
2. The antifungal activity of chlorhexidine gel increased over time, while the antifungal activity of calcium hydroxide decreased
3. Diapex Plus and Ledermix did not have any antifungal properties

## Figures and Tables

**Figure 2:**
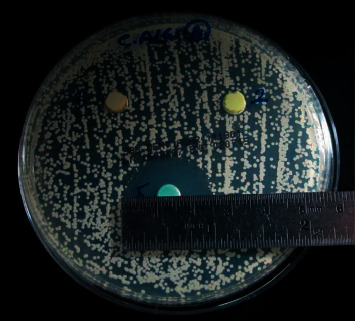
Measuring the inhibition zone using a metal ruler.

**Table 1:** Experimental root canal medicaments.

Materials	Composition	Manufacturer
Clorox Bleach Pen	0.5–2% sodium hypochlorite, 0.5–1.5% sodium silicate, 3–7% boehmite, 0.5–1.5% sodium sulfonate	Clorox, 1221 Broadway Oakland, CA 94612, USA
Consepsis scrub	2% chlorhexidine gel	Ultradent Products, USA
Ledermix	Demeclocycline calcium (30.21 mg/g), triamcinolone acetonide (10 mg/g), zinc oxide, silicon dioxide, calcium chloride, trolamine, sodium edetate, sodium sulphate	Riemser Pharma GMH, Germany
Metapaste	Calcium hydroxide, barium sulphate, polypropylene glycol	Meta Biomed Com. Ltd., South Korea
Diapex Plus	35–40% iodoform, 20–30% calcium hydroxide, polydimethylsiloxane	DiaDent Group International, South Korea

**Table 2:** Comparison between the mean diameters of growth inhibition zones (mm) developed by the tested medicaments against *C. albicans* at different time intervals.

Type of root canal medication	Mean values of inhibitory zones ± standard deviations (mm)			
	24 h	48 h	72 h	ANOVA (*P* value)
Sodium hypochlorite gel	52.2 ± 4.1^A1^	53.0 ± 4.8^A1^∗^^^*∗*^	51.8 ± 6.1^A1^	0.92
2% chlorohexidine gel	23.0 ± 1.1^B1^	24.9 ± 0.5^B2^	25.8 ± 0.7^B2^	*P* ≤ 0.001
Ledermix	0.0^C^	0.0^C^	0.0^C^	No comparison
Calcium hydroxide	20.7 ± 2.8^B1^	20.7 ± 2.8^B1^	16.3 ± 2.0^D2^	0.013
Diapex Plus	0.0^C^	0.0^C^	0.0^C^	No comparison
ANOVA (*P* value)	*P* ≤ 0.001	*P* ≤ 0.001	*P* ≤ 0.001	

^*∗*^Games–Howell post hoc test: different uppercase letters (columns) indicate a significant difference (*P* < 0.05) between the antifungal activity of the tested medicaments at each time interval. The different uppercase numbers (rows) indicate a statistically significant difference between the time intervals regarding the antifungal activity of each medicament.

## Data Availability

The data (measurements of inhibition zones) used to support the findings of this study will be available from Dr. Mohamed Elsayed at this e-mail elsayednada@yahoo.com for the researchers who meet the criteria for access to this data. The data can be requested after the publication of this article. However, requests for the data (6/12 months) after the publication of this article will be considered by the corresponding authors.
